# New alternative splicing variants of the ATXN2 transcript

**DOI:** 10.1186/s42466-019-0025-1

**Published:** 2019-07-03

**Authors:** Isabel Lastres-Becker, David Nonis, Joachim Nowock, Georg Auburger

**Affiliations:** 1 Experimental Neurology, Goethe University Medical Faculty, Building 89, 3rd floor, Theodor Stern Kai 7, 60590 Frankfurt am Main, Germany; 2 Present address: Department of Biochemistry, Faculty of Medicine, Universidad Autonoma of Madrid, Madrid, Spain

**Keywords:** ATXN2, Alternative splicing, Spinocerebellar Ataxia type 2, PolyQ expansion, ALS

## Abstract

**Background:**

Spinocerebellar ataxia type 2 (SCA2) is an autosomal dominant disorder with progressive degeneration of cerebellar Purkinje cells and selective loss of neurons in the brainstem. This neurodegenerative disorder is caused by the expansion of a polyglutamine domain in ataxin-2. Ataxin-2 is composed of 1312 amino acids, has a predicted molecular weight of 150-kDa and is widely expressed in neuronal and non-neuronal tissues. To date, the putative functions of ataxin-2 on mRNA translation and endocytosis remain ill-defined. Differential splicing with a lack of exons 10 and 21 was described in humans, and additional splicing of exon 11 in mice. In this study, we observed that the molecular size of transfected full-length wild-type ataxin-2 (22 glutamines) is different from endogenous ataxin-2 and that this variation could not be explained by the previously published splice variants alone.

**Methods:**

Quantitative immunoblots and qualitative reverse-transcriptase polymerase-chain-reaction (RT-PCR) were used to characterize isoform variants, before sequencing was employed for validation.

**Results:**

We report the characterization of further splice variants of ataxin-2 in different human cell lines and in mouse and human brain. Using RT-PCR from cell lines HeLa, HEK293 and COS-7 throughout the open reading frame of ataxin-2 together with PCR-sequencing, we found novel splice variants lacking exon 12 and exon 24. These findings were corroborated in murine and human brain. The splice variants were also found in human skin fibroblasts from SCA2 patients and controls, indicating that the polyglutamine expansion does not abolish the splicing.

**Conclusions:**

Given that Ataxin-2 interacts with crucial splice modulators such as TDP-43 and modulates the risk of Amyotrophic Lateral Sclerosis, its own splice isoforms may become relevant in brain tissue to monitor the RNA processing during disease progression and neuroprotective therapy.

## Background

The expansion of CAG trinucleotide repeats coding for polyglutamine domains in unrelated proteins causes at least nine autosomal dominantly inherited progressive neurodegenerative disorders, including Huntington’s disease and a number of spinocerebellar ataxias. Expanded polyglutamine domains cause neurotoxicity regardless of the specific protein context within which it resides. Nevertheless, the protein context does modulate the vulnerability threshold of specific neuron populations, as evidenced by the distinct clinical and pathological features of the various disorders [1–5]. Spinocerebellar ataxia type 2 (SCA2) is an autosomal dominant movement disorder caused by progressive degeneration of cerebellar Purkinje cells and spinal motor neurons, as well as selective loss of other neurons in the brainstem, spinal ganglia and thalamus [6–20]. The underlying protein, which is mutated in SCA2 and also acts as risk factor in the motor neuron degeneration of Amyotrophic Lateral Sclerosis (ALS), has been identified and named ataxin-2 (gene symbol *ATXN2*) [21–23].

The physiological function of ataxin-2 is unknown. It is a protein distributed diffusely throughout the cytoplasm that was at first claimed to be concentrated at the trans-Golgi network but later shown to reside at the rough endoplasmic reticulum or relocalize to stress granules [24–26]. It seems to be involved in RNA quality control, with its Pam2 motif mediating its direct association with the cytoplasmic poly(A)-binding protein (PABP) [27, 28], which functions in translation initiation and mRNA decay regulation. Direct interactions between ataxin-2 and target RNAs are mediated by two protein domains named Lsm and Lsm-AD, which associate preferentially with AU-rich transcript sequences and are highly conserved among ataxin-2 orthologues until yeast and plants [29–31]. The C-terminus of ataxin-2 can associate with RBFOX1 (also known as A2BP1 or ataxin-2 binding protein 1), a protein containing a ribonucleoprotein motif that is highly conserved among RNA-binding proteins and which was demonstrated to be important for alternative splicing, neural excitability and autism [32–34]. Another putative function is related to the endocytic machinery, where proline-rich-domains (PRD) in ataxin-2 associate with SH3 domains of endophilin-A and GRB2, connecting it to the plasma membrane receptors for epidermal growth factor and/or insulin, which influence different cellular pathways beginning with membrane curvature and vesicle internalization [35–37]. Interestingly, ataxin-2 is transcriptionally induced by starvation, and also the second member of this stress-response gene family in mammals, named ataxin-2-like (gene symbol *ATXN2L*), is upregulated at the mRNA and protein level by EGF signalling [38].

The ataxin-2 gene (NCBI Reference Sequence: NM_002973.4) is composed of 25 exons. The corresponding protein contains 1312 amino acids resulting in a predicted molecular weight of 150-kDa and is widely expressed in neuronal and non-neuronal human tissues [22]. Several studies have reported alternative splicing of ataxin-2. The splice variants were named type II in which exon 10 is lacking (present in human and mouse cDNAs) [39], type III lacking exon 10 and exon 11 (only seen in mouse) [39, 40] and type IV deficient in exon 21 [41]. Importantly, the alternative splicing of Ataxin-2 in neurons is under control of RBFOX2 and to be modulated by the SCA1 disease protein Ataxin-1 [42], while in oligodendroglial cells it is controlled by Quaking [43].

We observed transfected recombinant full length ataxin-2 cDNA to migrate at considerably higher size in Western-blots than endogenous full-length wild-type ataxin-2, with a size difference larger than explained by the known splice variants. Given the importance of alternative splicing in the pathogenesis of the pathogenesis of motor neuron degenerations like ALS and SMA, and given the importance of alternative splice events in the design of antisense-oligonucleotides in the treatment of SCA2 and ALS, we aimed to re-assess the main splicing events. In the present work, we report new splice variants of human and mouse ataxin-2 gene which lack exon 12 and 24.

## Materials and methods

### Cell culture and transfection procedure

HeLa, HEK293 and COS-7 cells were cultured in Dulbecco’s modified Eagle’s medium (DMEM, Gibco, Invitrogen) supplemented with 10% fetal bovine serum (PAA Laboratories GmbH) under standard conditions. Skin biopsies were taken from SCA2 patients and age matched controls after informed consent with approval of the local ethics committee, and fibroblasts from them were cultured in DMEM supplemented with 15% fetal bovine serum also under standard conditions. Two different lines of patient fibroblast were used, with different CAG expansion sizes: 38 and 41 CAG. HeLa cells were transfected with 10 μg DNA of pCMV-Myc-ataxin-2 plasmid using DreamFect (OZ Bioscience) according to the manufacturer’s instructions and cells were harvested 24 h after transfection. HEK293 and COS-7 cells were transfected using the Metafectene (Biontex) method and the Mirus reagent (Mirus Bio Corporation), respectively, and both harvested 36 h after transfection. Cells were washed twice with ice-cold PBS and suspended in lysis buffer (RIPA-buffer: 50 mM Tris-HCl, pH 8.0, 150 mM NaCl, 1 mM EDTA, 1 mM EGTA, 1% Igepal CA-630 (Sigma), 0.5% sodium deoxycholate, 0.1% SDS, a tablet of protease inhibitor cocktail (Roche), 1 mM PMSF, and 1 mM Na_3_VO_4_), lysed for 15 min at 4 °C and clarified by centrifugation at 16,000 g for 20 min. Mouse brain and cerebellum were homogenized in lysis buffer; after 15 min of incubation in lysis buffer, the lysate was clarified by centrifugation as above. All analyses were done at least in duplicate and reproduced.

### Western-blot

Lysates were resolved by 8% SDS-PAGE under reducing conditions and transferred to PVDF membranes. Blots were incubated (1:500) with the mouse monoclonal antibody from BD Transduction Laboratories against amino acids 713–904 of ataxin-2 (its specificity was previously established in *Atxn2*-KO mouse tissue [37]), and visualized using the ECL method (Pierce). All analyses were done at least in duplicate and reproduced.

### Reverse transcriptase-polymerase chain reaction (RT-PCR) for ataxin-2 transcripts

Total RNA from untransfected HeLa and HEK293 cells, human skin fibroblast and mouse brain or cerebellum was isolated using TRIZOL Reagent method (Invitrogen). The generation of cDNA from 5 μg RNA template was carried out with First-Strand cDNA Synthesis Kit (Amersham Bioscience). Human cDNA from cortex, cerebellum and liver were purchased from ClonTech Laboratories, Inc. 0ne μl of the first-strand mixture was added to 50 μl of the PCR mix containing 200 μM of dNTPs and 10 μM of sense and antisense primers (see Table 1). Amplified products were resolved on a 2% agarose-gel, purified using a QIAquick Gel Extraction kit (QIAGEN) and subjected to fluorescent DNA sequencing. All analyses were done at least in duplicate and reproduced.

**Table 1:** Sequence of the primers used to perform the PCR for the different fragments of ataxin-2 and GAPDH as loading control

RT-PCR	Primer name	Sequence 5’to 3´	Length (bp)
A	A-Forward	TGGGCAGAGGTCGAAACAGTAACA	24
	A-Reverse	TGCATCCCAGGGCTCCAGGTC	21
B	B-Forward	AATGTTCAGACTTTGTTGTGGTA	23
	B-Reverse	TCGGGTTGAAATCTGAAGTGTGAG	24
C	C-Forward	TGAGGGGCACAGCATAAACACT	22
	C-Reverse	CGTAGGAGATGCAGCTGGAATAGG	24
D	D-Forward	GGGGGAACGTGGTCATCAGTGGT	23
	D-Reverse	GGTTGCACGCCTGGGCTC	18
E	E-Forward	TCAGCCAAAGCCTTCTACTACCC	23
	E-Reverse	CATGTTGGCTTTGCTGCTGTC	21
F	F-Forward	CCCAAATTACCATACAACAAGGAG	24
	F-Reverse	GATGTGTTCATGACTTTCAAGG	22
GAPDH	Forward	TTCACCACCATGGAGAAGGC	20
	Reverse	GGCATCGACTGTGGTCATGA	20

### DNA sequencing and analysis

For the sequencing PCR we used the ABI PRISM Big Dye Terminator v3.0 Ready Reaction Cycle Sequencing Kit (Applied Biosystems) followed by ethanol/ sodium acetate purification. Samples were run in the sequencer ABI PRISM 377 (Applied Biosystems). Data were analysed using the DNASTAR programs Seqman and Megalign, with sequences being compared to the previously published sequence of human (NM_002973, gi:171543894) and mouse (NM_009125, gi: 124244103) ataxin-2.

## Results

### Endogenous ataxin-2 protein is smaller than the recombinant

**Fig. 1:**
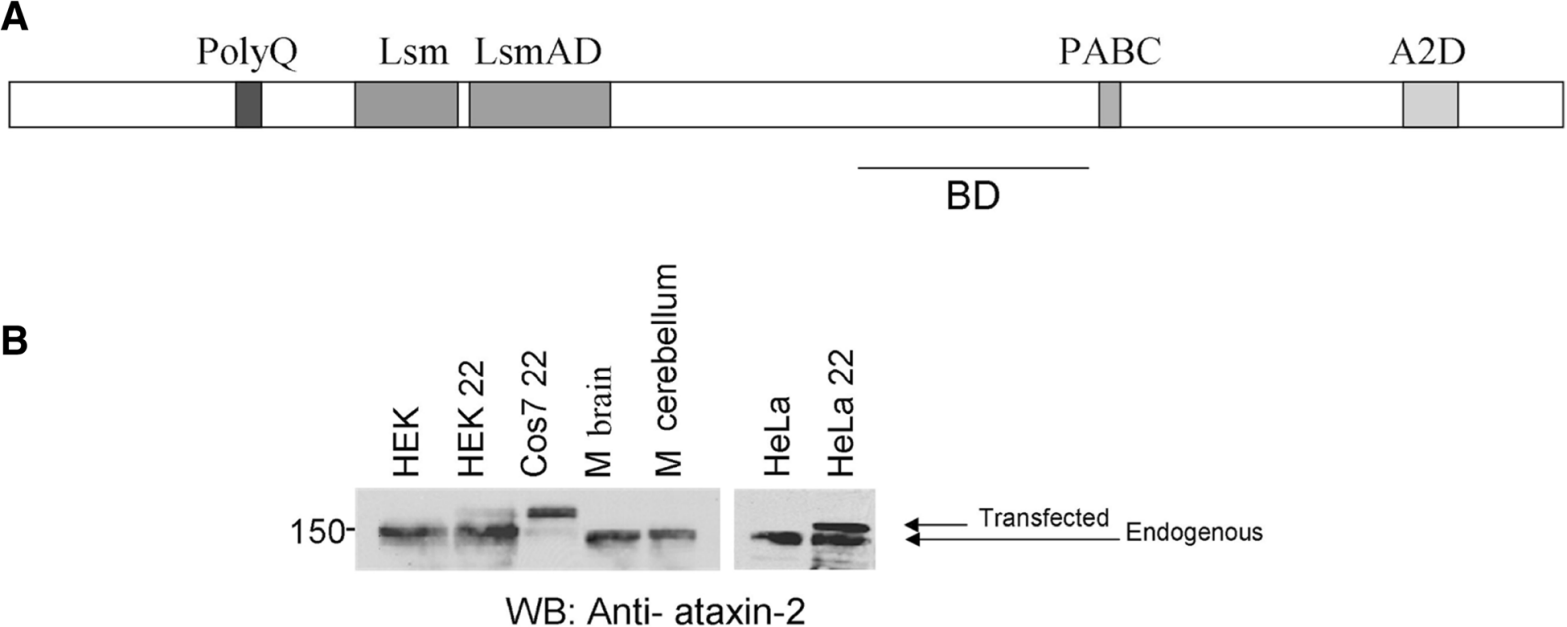
**a** Scheme of known ataxin-2 protein domains, with the site that is recognized by the monoclonal BD antibody. **b** Western-blot for ataxin-2 in different cell lines and mouse brain. Non-transfected and transfected (labelled 22 to indicate recombinant human wildtype ataxin-2 with a polyglutamine tract of 22 units) HeLa and HEK293, transfected COS-7 cell lysates as well as mouse brain and cerebellum lysates were loaded on a 8% SDS-PAGE gel. Detection of ataxin-2 in the transfected cells showed 2 bands, corresponding to the endogenous (lower band) and the transfected ataxin-2 protein (upper band), in comparison to the presence of a unique endogenous ataxin-2 band in the non-transfected cell lysates, in mouse brain and cerebellum extracts

We performed Western-blots from non-transfected cells, from different cell lines (monkey fibroblast from kidney cell line COS-7, human epithelial cervical cancer cell line HeLa and human embryonic kidney cell line HEK293) transfected with human wildtype ataxin-2 containing a polyglutamine domain of 22 units [pCMV-Myc-ataxin-2(22)], and also from mouse brain or cerebellum. Endogenous ataxin-2 was consistently smaller by about 15-kDa than the transfected protein whose recombinant episomal cDNA does not undergo any splicing (Fig. 1). There were no significant differences in the molecular weight of ataxin-2 between the different cell types (HeLa, HEK293, and COS-7). The mouse brain protein has a slightly smaller molecular weight than the corresponding protein in the various cell lines, probably due to the absence of the polyglutamine (polyQ) domain. The known splice variants lacking exon 10 with 210 bp and exon 21 with 54 bp cannot explain this size difference.

### Ataxin-2 presents two new splice variants

**Fig. 2:**
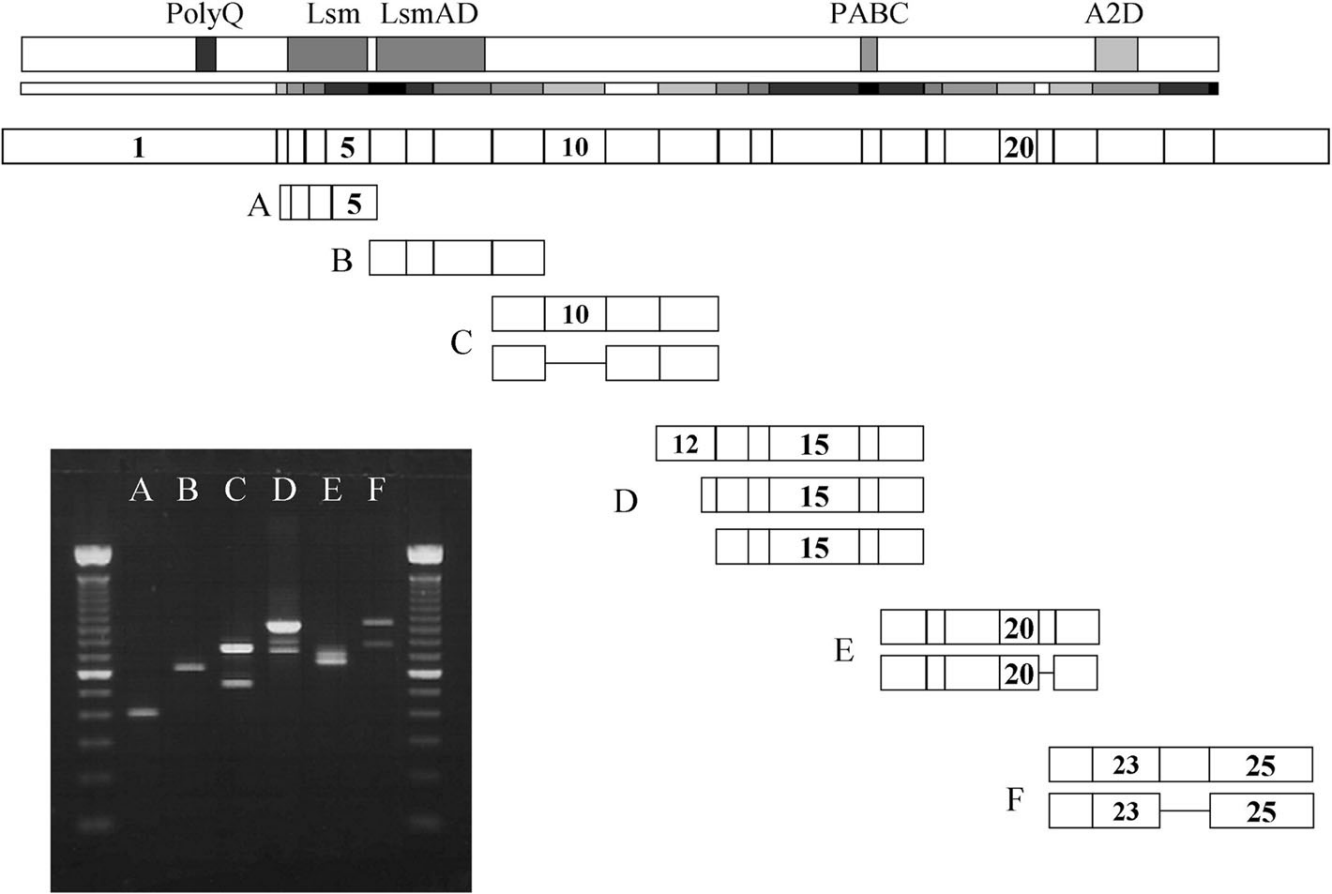
Above: Comparison of the structure of ataxin-2 at different levels. The first scheme presents ataxin-2 protein domains (A2D is an additional domain that is in common between ataxin-2 and ataxin-2-like, as reported by Meunier-C et al. 2002 J Biol Chem). The second scheme presents the exons that code for the protein. The third scheme presents the exon structure of ataxin-2. Below: For lanes **a** – **f** of the gel picture, each PCR band was sequenced and the shorter bands were shown to be deficient in different exons. Amplification fragments represent exons: A: 2–5 B: 6–9; C: 9–12; D: 12–17; E: 17–22; F: 22–25. RT-PCRs from HeLa RNA were resolved on a 2% agarose-gel, showing the different splice variants. The schematic PCR fragments to the right represent the sequencing results for full length PCR fragments and the shortened variants lacking different exons or exon fragments

**Fig. 3:**
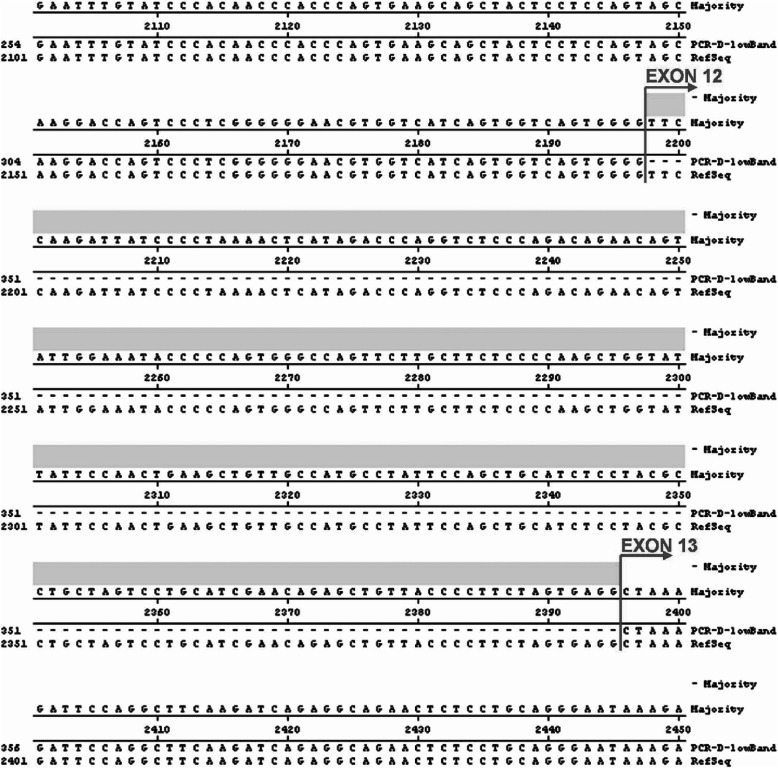
Alignment of the published human ataxin-2 cDNA sequence with the sequence obtained from the lower band of RT-PCR product D. Exon 12 was absent

**Fig. 4:**
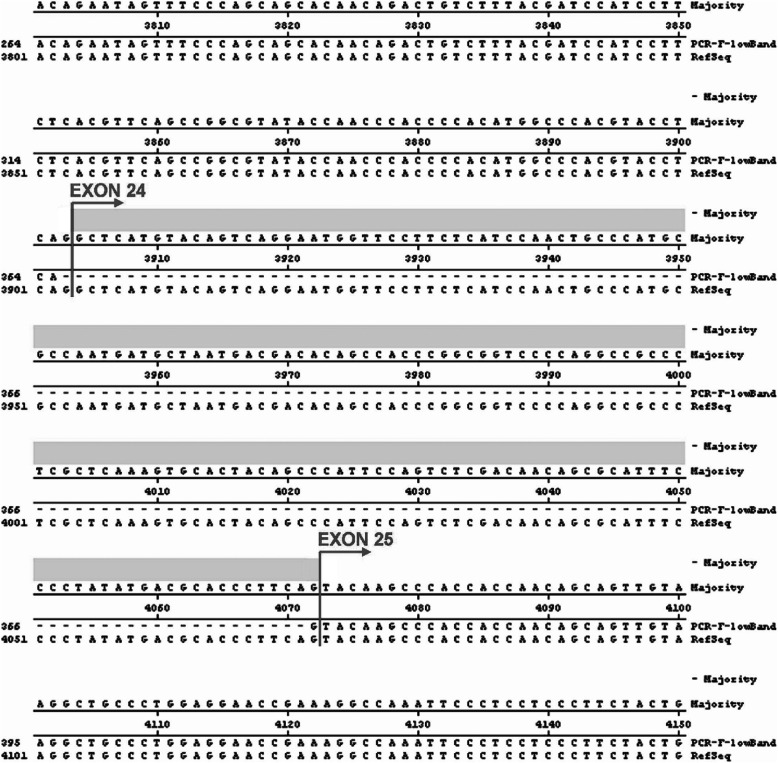
Alignment of the published human ataxin-2 cDNA sequence with the sequence from the lower band of RT-PCR product F. Exon 24 was absent

In order to investigate this discrepancy and the existence of additional variants, PCR primers were designed to amplify successive fragments of the ataxin-2 transcript from 5′ to 3′. In RNA from HeLa and HEK293 cells, and mouse brain or cerebellum, the sequencing of RT-PCR amplification bands confirmed the already published splice variants lacking exon 10 and exon 21 (Fig. 2). Two additional bands were amplified between exon 9 and exon 13 (Fig. 2, lane D), and one additional band at the C-terminus of the gene (Fig. 2, lane F), which have not been observed previously either in human (brain, spinal cord, cerebellum, heart and placenta) or in mouse (adult and whole embryos) tissue. Sequencing analysis of these bands, taking into account the GT-AG rule that predicts the junction between donor site and acceptor site [40], showed two full new exons, exon 12 (Fig. 3) and exon 24 (Fig. 4), as well as part of exon 12 (data not shown) to be lacking from the variant bands. We named these new splice variants type V (exon 12) and type VI (exon 24), to differentiate them from the known variants. To rule out any PCR artefacts, the same PCRs were performed with the plasmid DNA from pCMV-Myc-ataxin-2 (data not shown), yielding no alternative spliced products and confirming the selectivity of the PCR reaction.

### The presence of splicing variants is not dependent on the CAG repeat length

**Fig. 5:**
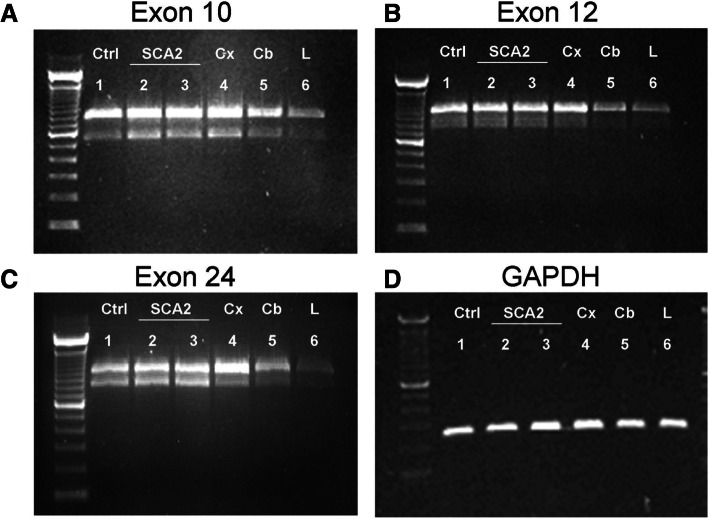
The presence of CAG repeat expansion does not abolish the appearance of splice variants. Lanes: 1-normal human fibroblasts (Ctrl); 2-SCA2 fibroblasts with 41 CAG repeats; 3-SCA2 fibroblasts with 38 CAG repeats. The three lines of fibroblasts presented splicing in exon 10 (**a**), exon 12 **b** and exon 24 (**c**). RNA loading was controlled in an additional gel with detection of GAPDH mRNA RT-PCR products (**d**). Ataxin-2 mRNA and splice variants in mouse cerebral cortex (lane 4), cerebellum (lane 5), liver (lane 6) showed the same exon pattern as above

The observed new splice variants were found in cells expressing normal or wild-type ataxin-2. To investigate whether the appearance of these splice variants is dependent on the polyglutamine length, we performed the same type of analysis using human skin fibroblasts from SCA2 patients with 38 and 41 polyglutamine expansions in comparison to control fibroblasts. The results showed that the already known splicing of exon 10 (Fig. 5a), as well as the newly observed splicing of exons 12 and 24 occur in both the control and the SCA2 patient mRNA (Fig. 5b and c), indicating that the splicing is not abolished by the CAG expansion in exon 1. This lack of influence of the CAG repeat length on the splicing was corroborated by the observation that all splice variants were present in mouse brain, where endogenous ataxin-2 contains a single glutamine instead of the polyglutamine domain.

### Ataxin-2 is present in different tissue in different amounts

It is well known that splice variants could be tissue specific, indicating different functions of the protein for the different tissues. To elucidate whether ataxin-2 splice variants are expressed in different tissues, we compared samples from human cerebral cortex, cerebellum and liver to the previous analysed. We observed that ataxin-2 full length and splice variants are expressed in human cell lines like HEK293, HeLa and human skin fibroblasts but also in human cerebral cortex and to a lesser extent in cerebellum and liver (Fig. 5a, b and c), although the amount used was the same (see GAPDH loading control). These data indicate that although ataxin-2 is expressed ubiquitously in all cells and tissues tested, the amount of expression differs, and so does the presence of the splice variants.

## Discussion

Our data demonstrate the existence of two new alternative splice variants of ataxin-2 transcript, lacking exon 12 and exon 24, expressed in two human cell lines, human skin fibroblasts as well as mouse and human brain. Interestingly, none of the alternatively spliced exons, neither the known exons 10 and 21, nor the newly observed exon 12 and exon 24, contain the established protein motifs described for ataxin-2 (Lsm according to UniProt entry Q99700 at amino acids 265–341 is encoded by exons 3–5, LsmAD at aa 409–476 is encoded by exons (6)-7–8, PAM2 at aa 909–926 is encoded by exon 16), but it is interesting to note that the putative PRD motif at aa 588–593 is encoded by the alternatively spliced exon 10. These data suggest that the alternatively spliced exons code for regulatory fine-tuning, while the invariably spliced exons code for the basic functions of ataxin-2 in RNA metabolism. This fine regulation of basic ataxin-2 function is not abolished by the polyglutamine expansion in patient cells, nor in mouse tissue where ataxin-2 contains one glutamine instead of the human polyglutamine domain. At present, it is impossible to elucidate the functional consequences of the new splicing variants, since functional assays for the physiological role of ataxin-2 are still lacking.

It cannot be decided yet, whether exon splicing, proteolytic events or an alternative translation start site lead to the unexpectedly short size of endogenous ataxin-2. Previous data demonstrating the splicing of exon 10 [40] and exon 21 [41], and the present data demonstrating the splicing of exon 12 and 24 consistently indicate that the full-length mRNA is always present in a significant amount. Western-blots detect an apparently single band representing the endogenous protein ataxin-2, indicating that size differences due to skipping of an individual exon would not be detectable in a 150 kDa protein, but the parallel skipping of several exons might become detectable. Ataxin-2 mRNA appears to be regulated with enormous complexity, given that (i) ataxin-2 exon 5 as circular RNA is induced 40-fold by Transforming Growth Factor beta under control of the transcription factor Quaking [44], (ii) ataxin-2 expression is complemented by a long antisense transcript [45] and (iii) ataxin-2 expression interacts with miRNA regulation [46]. Unfortunately, antibodies with specificity for the alternatively spliced domains of ataxin-2 do not exist, and their design and generation in future experiments would help greatly to identify the functional relevance for each of these ataxin-2 exons.

Recent research has also demonstrated ataxin-2 to act as risk factor in the motor neuron degenerative disease Amyotrophic Lateral Sclerosis (ALS), in RNA-mediated interaction with the splicing modulator TDP-43 [47–50]. Motor neuron degeneration due to TDP-43 aggregation pathology cannot be rescued by knockdown of TDP-43 itself, in view of the embryonal lethality of TDP-43 mutants, but the knockdown of the ALS risk factor ataxin-2 has been successful in preventing the neurodegenerative process [49]. For the design of such antisense-oligonucleotide-based neuroprotective therapies, the knowledge of alternative splicing patterns is crucial. Precise targeting of antisense oligonucleotides to correct splice events in the *SMN* gene is responsible for the current therapeutic breakthrough in the prevention of infantile motor neuron degeneration known as Spino-Muscular Atrophy (SMA). Thus, it will be very interesting to study the role of alternative splicing of ataxin-2 and of many TDP-43 dependent splice events throughout the global transcriptome in nervous tissues of ALS models or patients.

## Conclusion

Overall, the ataxin-2 transcript encodes a protein with putative functions in mRNA translation and endocytosis, which is consistently shorter than predicted full-length in different human cell lines as well as mouse and human brain. This observation may be partially explained by alternative splicing of exons 10, 12 (novel variant type V), 21 and 24 (type VI).
